# Comparison of two plasma p-tau217 assays to detect and monitor Alzheimer’s pathology

**DOI:** 10.1016/j.ebiom.2024.105046

**Published:** 2024-03-11

**Authors:** Joseph Therriault, Nicholas James Ashton, Ilaria Pola, Gallen Triana-Baltzer, Wagner Scheeren Brum, Guglielmo Di Molfetta, Burak Arslan, Nesrine Rahmouni, Cecile Tissot, Stijn Servaes, Jenna Stevenson, Arthur Cassa Macedo, Tharick Ali Pascoal, Hartmuth Christian Kolb, Andreas Jeromin, Kaj Blennow, Henrik Zetterberg, Pedro Rosa-Neto, Andrea Lessa Benedet

**Affiliations:** a Translational Neuroimaging Laboratory, McGill University Research Centre for Studies in Aging, Alzheimer’s Disease Research Unit, Douglas Research Institute, Le Centre Intégré Universitaire de Santé et de Services Sociaux (CIUSSS) de l'Ouest-de-l'Île-de-Montréal, Montréal, Québec H4H 1R3, Canada; b Department of Neurology and Neurosurgery, Faculty of Medicine, McGill University, Montreal, Quebec H3A 2B4, Canada; c Department of Psychiatry and Neurochemistry, Institute of Neuroscience and Physiology, The Sahlgrenska Academy, University of Gothenburg, Mölndal 6 431 41, Sweden; d Wallenberg Centre for Molecular Medicine, University of Gothenburg, Gothenburg 6 431 41, Sweden; e King’s College London, Institute of Psychiatry, Psychology and Neuroscience, Maurice Wohl Institute Clinical Neuroscience Institute, London SE5 9RT, UK; f NIHR Biomedical Research Centre for Mental Health and Biomedical Research Unit for Dementia at South London and Maudsley NHS Foundation, London SE5 8AF, UK; g Neuroscience Biomarkers, Janssen Research & Development, La Jolla, CA 92121, USA; h Department of Neurology and Psychiatry, University of Pittsburgh School of Medicine, Pittsburgh 15213, USA; i ALZpath. Inc, Carlsbad, CA 92008, USA; j Clinical Neurochemistry Laboratory, Sahlgrenska University Hospital, Mölndal 6 431 41, Sweden; k Department of Neurodegenerative Disease, UCL Institute of Neurology, Queen Square, London, UK; l UK Dementia Research Institute at UCL, London SE5 9RT, UK; m Hong Kong Center for Neurodegenerative Diseases, Clear Water Bay, Hong Kong 1512, China; n Wisconsin Alzheimer’s Disease Research Center, University of Wisconsin School of Medicine and Public Health, University of Wisconsin–Madison, Madison, WI 53792, USA

**Keywords:** Alzheimer’s disease, Blood biomarker, p-tau217, Comparison, Diagnosis

## Abstract

**Background:**

Blood-based biomarkers of Alzheimer’s disease (AD) have become increasingly important as scalable tools for diagnosis and determining clinical trial eligibility. P-tau217 is the most promising due to its excellent sensitivity and specificity for AD-related pathological changes.

**Methods:**

We compared the performance of two commercially available plasma p-tau217 assays (ALZpath p-tau217 and Janssen p-tau217+) in 294 individuals cross-sectionally. Correlations with amyloid PET and tau PET were assessed, and Receiver Operating Characteristic (ROC) analyses evaluated both p-tau217 assays for identifying AD pathology.

**Findings:**

Both plasma p-tau217 assays were strongly associated with amyloid and tau PET. Furthermore, both plasma p-tau217 assays identified individuals with AD vs other neurodegenerative diseases (ALZpath AUC = 0.95; Janssen AUC = 0.96). Additionally, plasma p-tau217 concentrations rose with AD severity and their annual changes correlated with tau PET annual change.

**Interpretation:**

Both p-tau217 assays had excellent diagnostic performance for AD. Our study supports the future clinical use of commercially-available assays for p-tau217.

**Funding:**

This research is supported by the 10.13039/100012479Weston Brain Institute, 10.13039/501100000024Canadian Institutes of Health Research (CIHR), 10.13039/100015569Canadian Consortium on Neurodegeneration in Aging, the 10.13039/100000957Alzheimer's Association, 10.13039/100009408Brain Canada Foundation, the 10.13039/501100000156Fonds de Recherche du Québec - Santé and the Colin J. Adair Charitable Foundation.

**Research in context**

**Evidence before this study**

Several recent observational studies have reported high performance of plasma phosphorylated tau (p-tau) for Alzheimer’s disease (AD), particularly p-tau217. As the availability of plasma p-tau217 tests begins to increase, it is important to understand if their differences in assay design and composition (e.g., targeting multiple or single phosphorylation sites) yield equivalent results.

**Added value of this study**

We showed that both commercially available plasma p-tau217 assays had excellent diagnostic performance for AD and had similarly strong associations with amyloid PET and tau PET and increased with disease severity measured by PET-based Braak staging.

**Implications of all the available evidence**

Findings suggest that both plasma p-tau217 assays may have sufficient clinical performance to be developed as diagnostic tests for use in memory clinics in the diagnostic workup of individuals with suspected neurodegenerative diseases and when determining which individuals are eligible for disease-modifying therapies. It is important that findings are further validated in populations with diverse backgrounds.

## Introduction

*In vivo* quantification of amyloid and tau pathologies using cerebrospinal fluid (CSF) and positron emission tomography (PET) has enabled the detection of Alzheimer’s disease (AD) in living humans.^1^^,^^2^ The recent emergence of blood-based biomarkers for AD promises to transform the *in vivo* diagnosis of AD by providing accessible information on the presence of pathological amyloid-β and tau proteins in the brain.^3^ Crucially, with the era of disease-modifying therapies for AD fast approaching,^4^^,^^5^ accessible and scalable biomarkers for AD pathology will be required.^6^ Specifically, the presence of amyloid pathology needs to be determined using biomarker investigations to determine whether a individual is eligible for anti-amyloid therapies.^7^^,^^8^ In addition, plasma biomarkers may be useful for identifying advanced tau pathology, as anti-amyloid therapies may be less effective in patients with advanced disease, as demonstrated in the recent phase II and phase III donanemab trials where amyloid-positive patients were stratified according to baseline tau burden.^5^^,^^9^

Phospho-tau217 (p-tau217) has emerged as one of the most promising blood-based biomarkers for AD based on its diagnostic performance,^10^ strong correlations with amyloid and tau pathologies,^11^, ^12^, ^13^, ^14^ and equivalence with established CSF biomarkers in head-to-head studies.^12^^,^^15^, ^16^, ^17^ However, biomarker assays targeting tau phosphorylation on Thr217 may differ because of their composition (e.g., use of antibodies targeting multiple or single phosphorylation sites) which may lead to distinct associations with pathology. Thus, there is a need to establish their associations with core biomarkers of AD and their comparative diagnostic performance. It is also important to note whether different plasma p-tau217 biomarkers are in agreement when detecting AD pathology *in vivo*, which will increase confidence in their future clinical use. Here, we aimed to compare two commercially available plasma p-tau217 immunoassays that differ in regard to their target specificity: the assay from ALZpath, targeting a single phosphorylation site (with minor p-tau181 and p-tau231 cross-reactivity),^17^ and the p-tau217 assay from Janssen (p-tau217+, additionally targeting p-tau212 and p-tau214)^18^ in individuals evaluated by dementia specialists and assessed with amyloid and tau PET.

## Methods

### Study participants

This investigation relies on a convenience sample drawn from the Translational Biomarkers of Aging and Dementia (TRIAD) cohort.^19^ This retrospective study specifically includes 294 participants who underwent amyloid and tau PET imaging, along with providing cross-sectional plasma samples for the measurement of both plasma p-tau217 assays (ALZpath and Janssen). Data was acquired between October 2017 and August 2022. In the TRIAD cohort clinical diagnosis is performed before biomarker data is collected, cognitively unimpaired (CU) participants have an Mini Mental State Examination (MMSE) score >24 and Clinical Dementia Rating (CDR) score of 0 and contains young (<30 years old) and older adults (>55 years old) individuals. Individuals with mild cognitive impairment (MCI) have a CDR score of 0.5, subjective and objective impairments in cognition, but preserved activities of daily living, in line with standard criteria.^20^ Patients with AD dementia met the diagnostic criteria of National Institute on Aging and the Alzheimer's Association criteria for Alzheimer's disease and had a CDR score ≥0.5.^21^ Patients diagnosed with dementia (being it of AD syndrome or not) and no evidence of amyloid pathology on PET were classified in this study as non-AD. The other diagnostic groups were also further classified in relation to their amyloid pathology status as described below. Exclusion criteria for the study included PET or magnetic resonance imaging (MRI) safety contraindications, recent major surgery, recent head trauma, or inadequately treated systemic conditions.

For a subset of 117 participants, plasma data in conjunction with tau PET data were available for at least one further time point from the baseline (summary information in Supplementary Table S1). For these participants biomarker’s annual change was estimated as described below.

### Ethics

All participants (or legal representatives) provided written informed consent and the study was approved by the Montreal Neurological Institute PET working committee and the Douglas Mental Health University Institute Research Ethics Board (MP-18-2017-157).

### Plasma and CSF p-tau217 assays

Blood samples were collected following previously described protocols.^22^ Plasma p-tau217+ Janssen concentration was measured using a Simoa assay developed by Janssen by scientists blinded to clinical and biomarker data as described previously.^15^ CSF and plasma ALZpath p-tau217 concentration was generated at the University of Gothenburg by scientists blinded to clinical and biomarker data as described previously.^17^

### Brain imaging

[^18^F]AZD4694 and [^18^F]MK6240 PET scans for amyloid and tau pathologies were acquired 40–70 min and 90–110 min post-injection, respectively. PET scans were acquired with a Siemens High Resolution Research Tomograph (Siemens Medical Solutions, Knoxville, Tennessee) and imaging data were processed, in conjunction with each individual’s MRI, as previously described, using the cerebellar grey matter and the inferior cerebellar grey matter as reference regions for Aβ and tau PET standardised uptake value ratio (SUVR) calculation, respectively. [^18^F]MK6240 PET images were skull-stripped before blurring to minimise spill-in from meningeal off-target binding. Amyloid-β positivity was determined if global [^18^F]AZD4694 SUVR was equal or greater than 1.55.^23^ For tau PET, a global index of tau pathology was given by the average SUVR in the temporal meta-region of interest (ROI) and tau positivity was defined as equal or greater than 1.24 SUVR.^24^ Regional tau PET was also quantified in the medial temporal regions and neocortical regions as previously published.^25^ Additional cut-offs for “low” or “advanced” tau accumulation were generated using 2.5 standard deviations of the mean medial temporal (>1.03) and neocortical (>1.06) SUVR of young participants, respectively. Participants were also categorised in PET-based Braak stages according to the topography of tau PET abnormality as described previously.^26^

### Statistics

R statistical software package (4.0.0) was used to perform non-imaging statistical analyses and statistical significance was set at *P* < 0.05, two-sided. Shannon-information value (*S*) is also reported for easier interpretability of the results, and it is equals to −log_2_(*P* value). For demographic descriptions, chi-square test compared sex proportions between groups and one-way analysis of variance (ANOVA) compared age between groups. Correlations between biomarkers were assessed with Spearman rank tests (confidence intervals (CI) at 95% were determined with 1000 bootstraps resampling) and, when necessary, correlation coefficients were compared using the ‘*cocor’* package.^27^ As mentioned above, participants were grouped according to diagnosis and amyloid statuses. Biomarker z-scores and fold mean were calculated using average values of the CU amyloid PET negative individuals as reference. For parametric statistical tests, fluid biomarkers were log transformed when appropriate. Analysis of covariance (ANCOVA) compared biomarker levels across groups adjusting for age and sex. Assumptions underlying ANOVA and ANCOVA models were evaluated with the package “*performance*”. The Bland–Altman comparison of the plasma assays was done using the ‘*blandr*’ package. For analyses of individual-level agreement between both plasma p-tau217 biomarkers, predefined thresholds for abnormality were used, as described previously for p-tau217+ (Janssen)^15^ and for p-tau217 (ALZpath).^17^ The area under the receiver operating characteristic curve (AUROC) assessed the biomarker accuracy to distinguish predefined statuses and 95% CI of sensitivities and specificities were also computed (Youden index). Voxelwise regression analyses were conducted on Rminc to evaluate the associations between the different plasma markers and *in vivo* amyloid PET and tau PET, adjusted for age and sex. One-tailed hypothesis tests with a type I error α < 0.05 were performed and Random-field theory^28^ was applied on the t-parametric maps to correct for multiple comparisons. Finally, annual biomarker changes were correlated to each other and, to this end, delta change (*Δ change = (follow-up – baseline)/time*) and percentage change (*% change= (Δ change/baseline)∗100*) were calculated.

### Role of funders

This research is supported by the Weston Brain Institute, Canadian Institutes of Health Research, Canadian Consortium of Neurodegeneration and Aging, the Alzheimer's Association, Brain Canada Foundation, the Fonds de Recherche du Québec – Santé and the Colin J. Adair Charitable Foundation. None of the funders had a role in the study design, data collection or data analyses.

## Results

**Fig. 1:**
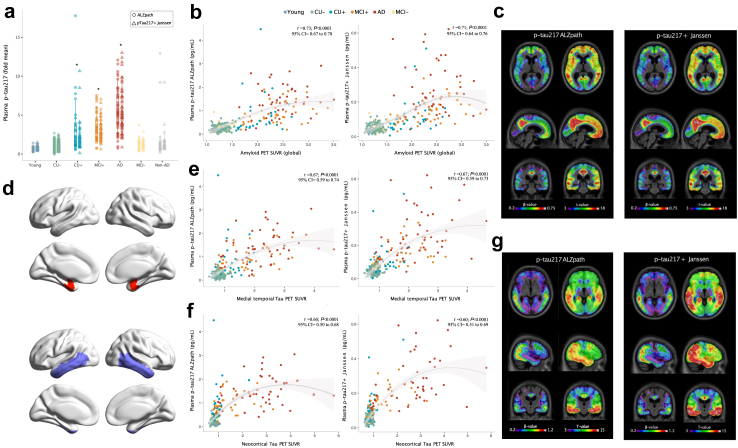
**Association of two plasma p-tau217 assays with amyloid PET and tau PET.** a: Plasma p-tau217 ALZpath and p-tau217+ Janssen in across diagnostic groups and amyloid PET status. The boxplots depict the median (horizontal bar), 25th to 75th percentiles (hinges) and whiskers indicate 10th and 90th percentiles. Group comparisons (presented in the Supplementary material) were computed with a one-way ANCOVA adjusting for age and sex. Tukey honest significant difference (HSD) test was used for the post hoc pairwise comparisons. The ∗ indicates, for each biomarker, the groups that are significantly different from CU- group. b: Correlation between plasma p-tau217 ALZpath and p-tau217+ Janssen with neocortical composite amyloid PET SUVR. The line represents the locally estimated scatterplot smoothing (LOESS; span = 1) regression and the shaded area shows the 95% confidence interval. c: Voxelwise association between plasma p-tau217 ALZpath and p-tau217+ Janssen with amyloid PET SUVR. d: Brain regions used for medial temporal (red) and neocortical (purple) tau PET ROI analyses. Brain regions were taken from Ossenkoppele et al.^25^ e: Correlation between plasma p-tau217 assays with medial temporal tau PET SUVR. The line represents the LOESS regression (span = 1) and the shaded area shows the 95% confidence interval. f: Correlation between plasma p-tau217 assays with neocortical temporal tau PET SUVR. The line represents the LOESS regression (span = 1) and the shaded area shows the 95% confidence interval. g: Voxelwise association between plasma p-tau217 assays with tau PET SUVR. N = 294.

We assessed concentrations of plasma p-tau217 using both assays in young adults, cognitively unimpaired (CU) amyloid-negative older adults, CU amyloid-positive older adults, amyloid-positive individuals with MCI, and amyloid-positive individuals with AD dementia, as well as in amyloid-negative individuals with MCI and individuals with non-AD neurodegenerative diseases (see Table 1 for clinical and demographic information). Concentrations of plasma p-tau217 rose with respect to clinical disease severity for both assays (ALZpath: *F* = 61.43, *P* < 0.0001, *S* > 13.28, ANCOVA; Janssen: *F* = 68.51, *P* < 0.0001, *S* > 13.28, ANCOVA; Fig. 1A, Supplementary Fig. S1 and Table S2 for group comparisons). Importantly, amyloid-negative individuals with MCI, and individuals with non-AD neurodegenerative diseases typically had low concentrations of plasma p-tau217 as measured with both assays, demonstrating high specificity for AD pathological changes. In addition, both assays strongly correlated with CSF p-tau217, as shown in the Supplementary Fig. S2 (ALZpath: *r* = 0.75, *P* < 0.0001, 95% CI = 0.66–0.81, *S* > 13.28, Spearman correlation; p-tau217+ Janssen: *r* = 0.76, *P* < 0.0001, 95% CI = 0.69–0.81, *S* > 13.28, Spearman correlation).

**Table 1:** Demographics and biomarker information.

	Young (N = 25)	CU− (N = 107)	CU+ (N = 32)	MCI+ (N = 42)	AD (N = 47)	MCI− (N = 19)	non-AD (N = 22)
Female sex	17 (68.0%)	63 (58.9%)	23 (71.9%)	26 (61.9%)	24 (51.1%)	9 (47.4%)	15 (68.2%)
Age, years	22.6 (1.44)	68.7 (9.03)	73.1 (8.17)	71.0 (5.73)	66.7 (7.78)	68.5 (11.0)	63.1 (9.86)
Race							
Asian	11 (44.0%)	0 (0%)	0 (0%)	0 (0%)	1 (2.1%)	1 (5.3%)	1 (4.5%)
Black or African American	0 (0%)	0 (0%)	0 (0%)	1 (2.4%)	0 (0%)	0 (0%)	0 (0%)
White	14 (56.0%)	107 (100%)	32 (100%)	40 (95.2%)	46 (97.9%)	18 (94.7%)	20 (90.9%)
Ethnicity							
Hispanic or Latino	0 (0%)	0 (0%)	1 (3.1%)	0 (0%)	0 (0%)	0 (0%)	1 (4.5%)
Not Hispanic or Latino	25 (100%)	107 (100%)	31 (96.9%)	42 (100%)	47 (100%)	19 (100%)	21 (95.5%)
MMSE, score	30.0 (0.20)	29.1 (1.01)	28.8 (1.24)	28.0 (1.80)	19.7 (6.74)	27.9 (1.60)	25.1 (6.23)
p-tau 217 ALZpath, pg/ml	0.16 [0.11, 0.20]	0.23 [0.16, 0.32]	0.48 [0.30, 0.91]	0.79 [0.53, 1.11]	1.35 [0.97, 1.86]	0.31 [0.19, 0.41]	0.21 [0.16, 0.35]
p-tau 217+ Janssen, pg/ml	0.04 [0.02, 0.04]	0.04 [0.03, 0.05]	0.08 [0.05, 0.11]	0.13 [0.10, 0.18]	0.23 [0.17, 0.35]	0.05 [0.04, 0.07]	0.05 [0.04, 0.07]
CSF p-tau 217, pg/ml	6.69 [5.33, 7.80]	10.4 [7.37, 16.1]	28.2 [24.1, 50.1]	45.6 [29.0, 77.7]	67.8 [48.3, 102]	11.4 [8.97, 17.4]	9.62 [6.56, 11.1]
Composite [^18^F]AZD4694 SUVR	1.17 [1.13, 1.21]	1.27 [1.21, 1.35]	2.01 [1.72, 2.27]	2.37 [2.09, 2.59]	2.43 [2.15, 2.68]	1.3 [1.24, 1.35]	1.23 [1.17, 1.36]
Med. Temp. [^18^F]MK6240 SUVR	0.74 [0.69, 0.81]	0.83 [0.74, 0.89]	1.04 [0.83, 1.35]	1.71 [0.95, 2.25]	2.30 [1.80, 2.76]	0.84 [0.73, 0.90]	0.80 [0.71, 0.93]
Neocortical composite [^18^F]MK6240 SUVR	0.84 [0.78, 0.90]	0.81 [0.75, 0.85]	0.84 [0.79, 0.94]	1.05 [0.87, 1.66]	2.80 [1.86, 3.32]	0.79 [0.75, 0.88]	0.78 [0.74, 0.90]
WMH	2.72 [1.88, 3.36]	3.83 [2.66, 6.17]	5.83 [3.34, 8.02]	5.35 [3.66, 9.84]	6.63 [4.65, 9.94]	7.00 [3.60, 11.6]	5.74 [3.57, 7.27]

Data are presented as count (%) or mean (Standard Deviation, SD), except for the fluid and imaging biomarkers which were given in median (IQR). No statistical difference was found in the proportion of sex types between groups (*Chi-squared* = 6.40, *P* = 0.37, *S* = 1.43). Age was statistically different between the groups, even when the Young group was not included in the model (ANOVA *F* = 4.80, *P* = 0.00032, S = 11.60; *post hoc* analysis, with Tukey HSD, showed that the CU+ (*P* = 0.00039, S > 13.32) and MCI+ (*P* = 0.0056, *S* = 7.48) groups are older than the non-AD group, whereas CU+ is older than the AD group (*P* = 0.013, *S* = 6.26)). The non-AD group comprises patients with clinical diagnoses such as Frontotemporal Dementia, Progressive Supranuclear Palsy, Alzheimer's Dementia Syndrome, and Mixed Dementia.

Next, we assessed the relationship between plasma p-tau217 assay concentrations [^18^F]AZD4694 SUVR, within the AD continuum (Fig. 1B). Both assays displayed very strong and equally good correlations with amyloid PET SUVR (ALZpath: *r* = 0.73, *P* < 0.0001, 95% CI = 0.67–0.78, *S* > 13.28, Spearman correlation; p-tau217+ Janssen: *r* = 0.71, *P* < 0.0001, 95% CI = 0.64–0.76, *S* > 13.28, Spearman correlation), which coefficients were not significantly different between each other (*P* = 0.46; 95% CI = −0.14–0.06, *S* = 1.12, Pearson and Filon’s z) (Fig. 1B). Voxelwise analyses also revealed similar topographical patterns of associations between plasma amyloid PET and plasma p-tau217 measured using both assays (Fig. 1C). When assessing the relationship between plasma p-tau217 status with amyloid PET in amyloid PET positive and negative groups separately, significant correlations were observed in the amyloid PET positive group for both assays (Supplementary Fig. S3). In the amyloid-negative group only p-tau217 ALZpath showed a statistically significant correlation with amyloid PET SUVR (ALZpath: *r* = 0.22, *P* = 0.0021, 95% CI = 0.07–0.37, *S* = 8.89, Spearman correlation; p-tau217+ Janssen: *r* = 0.08, *P* = 0.25, 95% CI = −0.06–0.23, *S* = 2, Spearman correlation), but no evidence of a difference between correlation coefficients was found (*P* = 0.43, 95% CI = −0.07–0.17, *S* = 1.21, Pearson and Filon’s z). We subsequently assessed the relationship of plasma p-tau217 assays with [^18^F]MK6240 tau PET, measured in both medial temporal and neocortical ROIs (Fig. 1D). Both assays had strong correlations with tau PET SUVR (ALZpath: *r*_med. temp._ = 0.67, *P* < 0.0001, 95% CI = 0.59–0.74, *S* > 13.28, Spearman correlation; p-tau217+ Janssen: *r*_med. temp._ = 0.67, *P* < 0.0001, 95% CI = 0.59–0.73, *S* > 13.28, Spearman correlation; ALZpath: *r*_neocort._ = 0.60, *P* < 0.0001, 95% CI = 0.50–0.68, *S* > 13.28, Spearman correlation; p-tau217+ Janssen: *r*_neocort_ = 0.60, *P* < 0.0001, 95% CI = 0.51–0.69, *S* > 13.28, Spearman correlation) (Fig. 1E and F). However, when looking at MCI+ and AD participants only, p-tau217+ (Janssen) better correlated with neocortical tau PET (*r*_neocort._ = 0.72, *P* < 0.0001, 95% CI = 0.59–0.81, *S* > 13.28, Spearman correlation) then did the ALZpath (*r*_neocort._ = 0.61, *P* < 0.0001, 95% CI = 0.45–0.73, *S* > 13.28, Spearman correlation) assay (*P* = 0.044; 95% CI = 0.004–0.23, *S* = 4.50) (Supplementary Fig. S4). Voxelwise analyses also revealed similar topographical patterns of associations between plasma tau PET and plasma p-tau217 measured using both assays, with slightly stronger associations observable for plasma p-tau217+ Janssen (Fig. 1G).

**Fig. 2:**
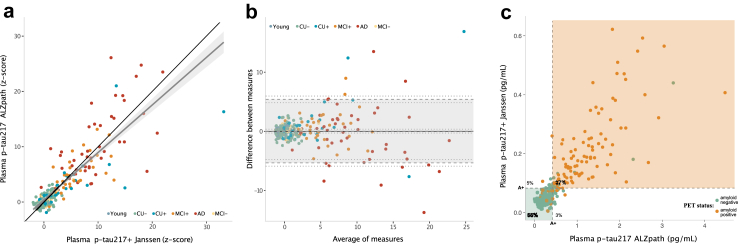
**Head-to-head comparison of plasma p-tau217 and p-tau217+.** a: Linear regression between plasma p-tau217 ALZpath and p-tau217+ Janssen (z-scored); the grey line represents the linear regression between the biomarkers with its 95% confidence interval (shaded area) whereas in black is the identity line. b: Bland–Altman comparison of plasma p-tau217 and p-tau217+ concentrations indicating high levels of agreement between both assays and few individuals with biomarker concentrations outside the limits of agreement. c: Excellent (92%) within-subject agreement between plasma p-tau217+ (Janssen) and plasma p-tau217 (ALZpath) statuses. The Scatter plot shows the distribution of plasma p-tau217 concentrations across assays and cut-offs. The dashed lines indicate the cut-off values for of each of the plasma p-tau217 assays to indicate amyloid PET status (A+). The shaded areas represent the range of values in which individuals would be classified as amyloid PET negative (green), amyloid PET positive (orange) based on the plasma assays cut-offs. The values displayed within the plot show the percentage (%) agreement between plasma assays. The dots are coloured according to the individual status on the PET imaging classification, as presented in the legend in the figure. N = 294.

Subsequently, we assessed the relationship of both plasma biomarker assays to each other. Fig. 2A displays a scatterplot of z-scored p-tau217 ALZpath concentration with respect to p-tau217+ Janssen concentration. We observed a strong correlation between both plasma p-tau217 assays (*r* = 0.85, *P* < 0.0001, 95% CI = 0.79–0.88, *S* > 13.28, Spearman correlation). The black line indicates the line of identity. A Bland–Altman plot representing the agreement between p-tau217 biomarkers is displayed in Fig. 2B. Datapoints outside the limits of agreement were more common at higher concentrations than when the mean of both plasma biomarkers was close to 0, indicating that higher concentrations of plasma p-tau217 are less likely to agree between measurement methods. A similar number of datapoints was observed outside of the upper and lower limits of agreement, and a test for bias was not significant (*t* = 0.28; *P* = 0.77, *S* = 0.37, Bland–Altman). We subsequently assessed the individual-level agreement of both p-tau217 assays (Fig. 2C). There was agreement between both plasma p-tau217 assays in 92% of cases (37% Janssen+/ALZpath+ and 55% Janssen-/ALZpath-) and disagreement in 8% of cases (5% Janssen+/ALZpath- and 3% Janssen-/ALZpath+). Furthermore, both assays had excellent agreement with each other when identifying amyloid PET positive individuals and when identifying individuals with biomarker-defined AD (Supplementary Fig. S5).

**Fig. 3:**
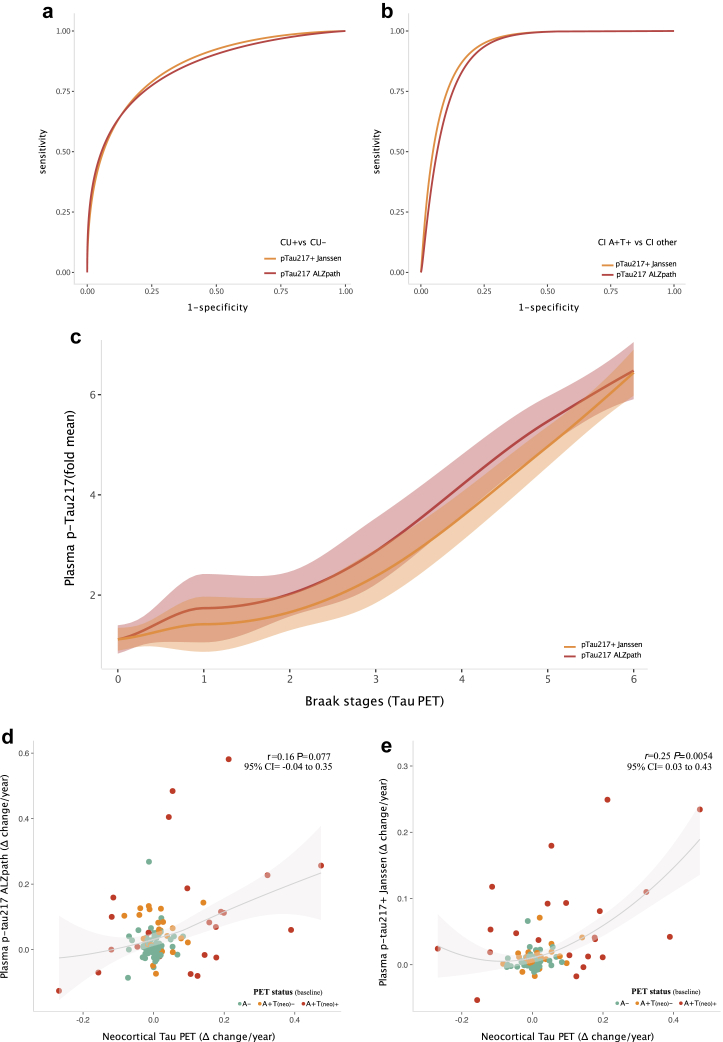
**Plasma p-tau217 assays identify biological AD *in vivo*, track AD severity and correlate with tau PET progression.** a: ROC curves for plasma p-tau217 ALZpath and p-tau217+ Janssen differentiating among amyloid PET positive and negative individuals without cognitive impairment (AUC_ALZpath_: 0.84, 95% CI = 0.76–0.92; AUC_Janssen_: 0.86, 95% CI = 0.79–0.93; Delong’s comparison test unveiled a *P* = 0.58, 95% CI = −0.04 to 0.08, S = 0.78). b: ROC curves for plasma p-tau217 ALZpath and p-tau217+ Janssen for the identification of biological AD (amyloid PET and tau PET positivity) in individuals with cognitive impairment (AUC_ALZpath_: 0.91, 95% CI = 0.86–0.96; AUC_Janssen_: 0.92, 95% CI = 0.88–0.96; Delong’s comparison test unveiled a *P* = 0.50, 95% CI = −0.02 to 0.04, S = 1). c: Plasma p-tau217 ALZpath and p-tau217+ Janssen rise with AD severity measured using PET-based Braak staging. The line represents the locally estimated scatterplot smoothing (LOESS) regression and the shaded area shows the 95% confidence interval. d and e: Correlation of annual change in plasma p-tau217 assays with annual change in tau PET (meta-ROI). The line represents the LOESS regression (span = 1) and the shaded area shows the 95% confidence interval. Using the PET status, amyloid-negative participants are here labelled as A−; individuals amyloid-positive and neocortical tau-negative are labelled as A + T(neo)−; and individuals amyloid-positive and neocortical tau-positive are labelled as A + T(neo)+. N = 294.

Next, we evaluated the diagnostic performance of both plasma biomarkers for identifying amyloid PET positivity in cognitively unimpaired individuals, as well as identifying biological AD (amyloid PET and tau PET positivity) in cognitively impaired individuals evaluated by dementia specialists (Fig. 3A and B; Supplementary Table S3). Both plasma p-tau217 assays also demonstrated excellent performance at identifying amyloid PET positivity in all individuals (ALZpath: AUROC = 0.92, 95% CI = 0.89–0.95, ROC; p-tau217+ Janssen: AUROC = 0.92, 95% CI = 0.89–0.95, ROC). Furthermore, both assays had excellent performance for identifying biological AD (amyloid PET and tau PET positivity) in cognitively impaired individuals (ALZpath: AUROC = 0.91, 95% CI = 0.86–0.96, ROC; p-tau217+ Janssen: AUROC = 0.92, 95% CI = 0.88–0.96, ROC) and in the whole sample (ALZpath: AUROC = 0.95, 95% CI = 0.93–0.97, ROC; p-tau217+ Janssen: AUROC = 0.96, 95% CI = 0.94–0.98, ROC). We also observed that both plasma p-tau217 assays yielded increased values according to AD severity measured using PET-based Braak staging (ALZpath: *F* = 64.98, *P* < 0.0001, *S* > 13.28, ANCOVA; p-tau217+ Janssen: *F* = 75.63, *P* < 0.0001, *S* > 13.28, ANCOVA; Fig. 3C) with sharper increases appearing at stage III. Finally, we tested the ability of both plasma assays to track longitudinal disease progression measured using tau PET. We observed that both plasma p-tau217 assays show a similar association with annual tau PET change in the neocortex (ALZpath: *r* = 0.16, *P* = 0.077, 95% CI = −0.04 to 0.35, *S* = 3.69, Spearman correlation; p-tau217+ Janssen: *r* = 0.25, *P* = 0.0054, 95% CI = 0.03–0.43, *S* = 7.53, Spearman correlation; correlation comparison: *P* = 0.31; 95% CI = −0.08 to 0.26, *S* = 1.68, Pearson and Filon’s z; Fig. 3D and E). Furthermore, both annual and percentage change of plasma p-tau217+ Janssen and plasma p-tau217 ALZpath were highly correlated (Supplementary Fig. S6).

## Discussion

This study compared two commercially available research use only (RUO) assays for plasma p-tau217 head-to-head, assessing their relationships with amyloid PET and tau PET, as well as their diagnostic performance for AD. Both assays had similar and strong associations with neocortical amyloid PET. Furthermore, both plasma assays had essentially identical diagnostic performance for amyloid PET positivity in asymptomatic individuals, as well as for amyloid PET and tau PET positivity in cognitively impaired individuals. We observed that the p-tau217+ Janssen assay had mildly stronger associations with neocortical tau PET in individuals with symptomatic AD than did p-tau217 ALZpath. Taken together, our study provides evidence that new commercial RUO assays for plasma p-tau217 may have high diagnostic performance for AD.

Plasma p-tau217 biomarkers have shown strong discriminative accuracy for AD, as well as strong relationships between amyloid and tau pathologies in several studies,^11^, ^12^, ^13^, ^14^ which suggests that p-tau217 has emerged as a strong candidate for future prospective clinical implementation studies.^29^ This head-to-head study provides evidence that different p-tau217 assays may be suitable for evaluating diagnostic performance and disease monitoring in AD. Future studies are required to evaluate whether additional p-tau biomarkers, which have different associations with key pathological features of AD,^11^^,^^14^^,^^30^^,^^31^ provide additional diagnostic or prognostic information in clinical settings.^32^

Plasma concentrations of both p-tau217 assays increased sequentially from CU amyloid negative individuals to CU amyloid positive individuals, to amyloid positive MCI to amyloid positive individuals with AD dementia. Notably, the particularly low concentrations of plasma p-tau217 ALZpath and p-tau217+ Janssen in young adults, as well as amyloid negative individuals highlight the excellent specificity of p-tau217 for AD pathological change. While more work is needed to understand the interpretation of plasma biomarkers at the individual-level, the increase of plasma p-tau217 in asymptomatic individuals with elevated amyloid pathology suggests that routine evaluation of plasma p-tau217 may become a potential screening strategy for early AD pathology, provided therapeutic interventions are effective in this population. Mild elevations of plasma p-tau217 in early clinical disease could be a trigger for confirmatory amyloid PET or CSF testing before initiating AD disease-modifying treatments, provided disease-modifying therapies demonstrate efficacy in presymptomatic AD.

Both plasma p-tau217 biomarkers performed excellently in differentiating the etiology of the underlying disease in individuals with cognitive impairment. Accurately diagnosing the etiology of cognitive impairment is crucial in light of evidence that AD can present clinically as a variety of non-amnestic syndromes.^33^, ^34^, ^35^ Building on studies showing high performance of plasma p-tau217 in identifying individuals with AD in memory clinic centres,^16^ and equivalent performance between plasma p-tau217 and CSF assessments of AD,^12^^,^^15^ the present findings highlight the suitability of plasma p-tau217 for future clinical implementation studies. Due to the large differences in concentrations of plasma p-tau217 in individuals with symptomatic AD (i.e., who have advanced disease) vs symptomatic individuals with other neurodegenerative diseases where p-tau217 is normal,^13^ plasma p-tau217 may be particularly well-suited for differential diagnosis in clinically symptomatic populations. Crucially, both assays had high individual-level agreement when identifying patients with biological AD, suggesting that plasma p-tau217 abnormality is a reliable signature of AD pathology. These finding suggests that plasma p-tau217 may have sufficient clinical performance to be developed as diagnostic tests for use in memory clinics in the diagnostic workup of individuals with suspected neurodegenerative diseases and when determining which individuals are eligible for disease-modifying therapies.^7^

The correlation of plasma p-tau217 biomarkers annual changes with tau PET annual changes suggests that plasma p-tau biomarkers may be beneficial for monitoring disease progression in clinical trials.^36^ Although substantial variability was observed between individual-level tau PET change and individual-level plasma p-tau217 change, their overall association suggests that changes in plasma p-tau217 may be a useful secondary outcome in clinical trials targeting AD pathology, as was performed in the phase II donanemab trial^37^ and the lecanemab phase III trial.^4^ However, because p-tau217 is closely associated with both amyloid and tau pathologies,^11^^,^^14^ these biomarker signals should be interpreted with caution. Indeed, the recent phase III donanemab trial demonstrated substantial decreases in plasma p-tau217 with anti-amyloid treatment, but no differences in longitudinal tau PET change was observed between donanemab and placebo groups.^5^

Our study has limitations. As blood biomarkers move closer to clinical use, a better understanding of their performance in real-world and diverse populations is required. Regrettably, primarily due to the homogeneity of the sample regarding race, the lack of diversity in this aspect is an acknowledged shortcoming, and we recognise the importance of exploring potential variations in biomarker performance across different racial or ethnic groups. While blood-biomarker performance in real-world populations is generally quite favourable,^38^ the influence of systemic medical conditions such as chronic kidney disease,^39^ which was not accounted for in the current report, needs to be further elucidated; recent evidence suggests that plasma p-tau217/tau ratios may help in these cases.^40^ The influence of pre-analytical variability in such real-world situations also needs to be investigated, since the current study, being a single-center study, allowed for a more stringent control of blood and cerebrospinal fluid sample handling, ensuring greater consistency and stability compared to real-world multicenter studies. However, before plasma p-tau measurement can be widely used in clinical settings, it is crucial to gain a comprehensive understanding of how the variability and biases in blood measurements can impact the quantification of plasma p-tau. Furthermore, prospective studies with pre-defined thresholds are required to further evaluate the accuracy and utility of plasma biomarkers in a way that is closer to how they will be expected to perform in routine clinical settings.^41^

In conclusion, the present study compared two recently developed commercial RUO p-tau217 assays head-to-head. Both assays had high correlations with amyloid PET and tau PET, and their read-outs rose with AD severity. Finally, both the ALZpath p-tau217 and Janssen p-tau217+ assays had excellent performance for detecting amyloid PET positivity in asymptomatic individuals, and for detecting individuals with biological AD among a sample of cognitively impaired individuals.

## Contributors

JT, NJA, PRN, KB, HZ and ALB created the concept and design of the study. Data acquisition and analysis was performed by JT, NJA, IP, WSB, GDM, BA, NR, CT, SS, JS, ACM, TP and ALB. JT, NJA, GTB, HCK, AJ and ALB contributed to the sample selection/and or interpretation of the data. JT, NJA and ALB verified the underlying data. JT, NJA and ALB drafted the manuscript and all authors revised. All authors read and approved the final manuscript.

## Data sharing statement

This study includes no data deposited in external repositories. Anonymised data can be shared upon reasonable request from a qualified academic investigator, for the sole purpose of replicating procedures and results presented in the article, as long as data transfer agrees with local legislation and with the local Ethical Review Board of the cohort, which must be regulated in a material/data transfer agreement.

## Declaration of interests

Joseph Therriault has received consulting fees from the Neurotorium educational platform, outside of the scope of this work. Nicholas J. Ashton has served as consultant for Quanterix and has given lectures in symposia sponsored by Lilly, Quanterix and Biogen. Gallen Triana-Baltzer and Hartmuth Kolb are employees of, and have stocks from, Janssen R&D, who pays their travel-related expenses. They have been also granted and filled patent applications (US20190271710A1 and JAB7064USPSP2). Andreas Jeromin is an employee of, and has stocks from, Alzpath Inc. Pedro Rosa-Neto has served at scientific advisory boards and/or as a consultant for Roche, Novo Nordisk, Eisai, and Cerveau radiopharmaceuticals. Henrik Zetterberg has served at scientific advisory boards and/or as a consultant for Abbvie, Acumen, Alector, Alzinova, ALZPath, Annexon, Apellis, Artery Therapeutics, AZTherapies, Cognito Therapeuthics, CogRx, Denali, Eisai, Nervgen, Novo Nordisk, Optoceutics, Passage Bio, Pinteon Therapeutics, Prothena, Red Abbey Labs, reMYND, Roche, Samumed, Siemens Healthineers, Triplet Therapeutics, and Wave, has given lectures in symposia sponsored by Cellectricon, Fujirebio, Alzecure, Biogen, and Roche, and is a co-founder of Brain Biomarker Solutions in Gothenburg AB (BBS), which is a part of the GU Ventures Incubator Program (outside submitted work). Kaj Blennow has served as a consultant, at advisory boards, or at data monitoring committees for Acumen, Abcam, ALZpath, AriBio, Axon, BioArctic, Biogen, Eisai, JOMDD/Shimadzu, Julius Clinical, Lilly, MagQu, Novartis, Ono Pharma, Pharmatrophix, Prothena, Roche Diagnostics, and Siemens Healthineers, and is a co-founder of Brain Biomarker Solutions in Gothenburg AB (BBS), which is a part of the GU Ventures Incubator Program, outside the work presented in this paper. All other authors report no disclosures.
