# Supplementary material for: Comparison of two plasma p-tau217 assays to detect and monitor Alzheimer’s pathology
Source: eBioMedicine. 2024 Mar 11;102:105046. doi: 10.1016/j.ebiom.2024.105046 (PMC10943661; doi:10.1016/j.ebiom.2024.105046)
Supplement: Supplementary Figs. S1–S6 and Tables S1–S3 [file mmc1.docx]

**SUPPLEMENTARY MATERIAL**

**Comparison of two plasma p-tau217 assays to detect and monitor Alzheimer’s pathology**

Joseph Therriault PhD^1,2^, Nicholas James Ashton PhD^3,4,5,6^, Ilaria Pola MSc^3^, Gallen Triana-Baltzer ^7^, Wagner Scheerem Brum^3^, Guglielmo Di Molfetta MSc^3^, Burak Arslan MD^3^, Nesrine Rahmouni MSc^1,2^, Cecile Tissot PhD^1,2^, Stijn Servaes PhD^1,2^, Jenna Stevenson BA^1,2^, Arthur Cassa Macedo MD^1,2^, Tharick Ali Pascoal MD PhD^8^, Hartmuth Christian Kolb PhD^7^, Andreas Jeromin PhD^9^, Kaj Blennow MD PhD^3,10^, Henrik Zetterberg MD PhD ^3,10-14^, Pedro Rosa-Neto MD PhD^1,2^ and Andrea Lessa Benedet PhD^3^

**Table of contents**

**Supplementary tables**

- **Supplementary Table. S1:** Demographic and biomarker information of the subset of participants with available longitudinal data.
- **Supplementary Table. S2:** Comparison of plasma p-tau 217 levels across groups*: post hoc* results.
- **Supplementary Table. S3:** ROC analyses.

**Supplementary figures**

- **Supplementary Fig. S1:** Box-plot showing plasma p-tau 217 levels across groups displaying raw values on pg/mL.
- **Supplementary Fig. S2:** Correlation of plasma p-tau217 assays with CSF p-tau 217.
- **Supplementary Fig. S3:** Correlation of plasma p-tau217 assays with amyloid PET uptake in amyloid PET positive and amyloid PET negative groups separately.
- **Supplementary Fig. S4:** Associations between plasma p-tau217 and tau PET in cognitively impaired amyloid PET positive individuals.
- **Supplementary Fig. S5:** Individual-level agreement of plasma p-tau217 assays for identifying amyloid PET positivity and biomarker-defined AD.
- **Supplementary Fig. S6:** Correlation of annual change of plasma p-tau217+ and plasma p-tau217

**Table. S1:** Demographic and biomarker information of the subset of participants with available longitudinal data.

|  | **Young (N=5)** | **CU- (N=52)** | **CU+ (N=16)** | **MCI+ (N=23)** | **AD (N=13)** | **MCI- (N=8)** |
| --- | --- | --- | --- | --- | --- | --- |
| **Female sex, n (%)** | 4  (80.0%) | 33 (63.5%) | 10 (62.5%) | 12 (52.2%) | 7  (53.8%) | 5  (62.5%) |
| **Age, years (BL)** | 22.67 [21.86, 25.15] | 68.30 [66.47, 74.23] | 72.50 [69.82, 74.90] | 72.86 [66.86, 76.18] | 62.31 [56.64, 70.04] | 74.80 [69.15, 76.01] |
| **p-tau217 ALZpath (BL),**  **pg/mL** | 0.06  [0.06, 0.10] | 0.12  [0.09, 0.16] | 0.23  [0.16, 0.43] | 0.46  [0.32, 0.73] | 0.73  [0.65, 1.14] | 0.13  [0.09, 0.20] |
| **p-tau217 ALZpath (FU),**  **pg/mL** | 0.10  [0.10, 0.10] | 0.13  [0.10, 0.18] | 0.37  [0.27, 0.53] | 0.52  [0.35, 0.89] | 0.93  [0.67, 1.30] | 0.16  [0.14, 0.22] |
| **p-tau217+ Janssen (BL)**  **pg/mL** | 0.03  [0.03, 0.04] | 0.04  [0.03, 0.05] | 0.09  [0.05, 0.13] | 0.13  [0.09, 0.17] | 0.29  [0.19, 0.44] | 0.05  [0.04, 0.08] |
| **p-tau217+ Janssen (FU)**  **pg/mL** | 0.04  [0.04, 0.04] | 0.04  [0.04, 0.06] | 0.11  [0.07, 0.18] | 0.15  [0.11, 0.21] | 0.43  [0.24, 0.63] | 0.07  [0.05, 0.08] |
| **Composite [^18^F]AZD4694 SUVR (BL)** | 1.17  [1.14, 1.18] | 1.27  [1.21, 1.33] | 2.02  [1.63, 2.25] | 2.37  [2.13, 2.56] | 2.10  [2.01, 2.44] | 1.31  [1.30, 1.44] |
| **Med. Temp. [^18^F]MK6240 SUVR (BL)** | 0.85  [0.79, 0.95] | 0.85  [0.75, 0.92] | 0.88  [0.79, 1.32] | 1.87  [1.17, 2.45] | 2.33  [1.68, 2.50] | 0.85  [0.76, 0.93] |
| **Med. Temp. [^18^F]MK6240 SUVR (FU)** | 0.76  [0.65, 0.98] | 0.82  [0.74, 0.93] | 0.91  [0.78, 1.49] | 1.99  [1.35, 2.50] | 2.05  [1.57, 2.32] | 0.82  [0.76, 0.89] |
| **Neocortical composite [^18^F]MK6240 SUVR (BL)** | 0.85  [0.83, 0.92] | 0.81  [0.75, 0.85] | 0.80  [0.78, 0.91] | 1.10  [0.90, 1.45] | 2.80  [2.06, 3.59] | 0.76  [0.74, 0.82] |
| **Neocortical composite [^18^F]MK6240 SUVR (FU)** | 0.80  [0.79, 0.94] | 0.81  [0.75, 0.85] | 0.82  [0.77, 0.89] | 1.29  [0.99, 1.61] | 2.87  [2.14, 3.31] | 0.76  [0.71, 0.83] |
| **Difference between time points (years)** | 2.13  [2.11, 2.38] | 1.91  [1.23, 2.20] | 2.12  [1.25, 2.40] | 2.06  [1.24, 2.41] | 1.45  [1.12, 1.92] | 1.12  [0.98, 2.24] |
|  |  |  |  |  |  |  |

Data are presented as count (%) or median (IQR).

*Abbreviations: AD, Alzheimer’s disease dementia; BL, Baseline assessment; CSF, cerebrospinal fluid; CU−, amyloid-negative cognitively unimpaired; CU+, amyloid-positive cognitively unimpaired; FU, follow-up assessment; IQR, interquartile range; MCI+, amyloid-positive mild cognitive impairment; MCI-, amyloid-negative mild cognitive impairment; Med. Temp., Medial Temporal; p-tau, phosphorylated tau; SUVR, Standard uptake value ratio.*

**Table. S2:** Comparison of plasma p-tau217 levels across groups*: post hoc* results.

|  | **p-tau217 ALZpath** | | | **p-tau217+ Janssen** | | |
| --- | --- | --- | --- | --- | --- | --- |
| **Groups** | **Estimate** | **T value** | **Adj. *P* value** | **Estimate** | **T value** | **Adj. *P* value** |
| CU- - Young | -0.04 | -0.40 | 0.99 | 0.049 | 0.531 | 0.99 |
| CU+ - Young | 0.32 | 2.68 | 0.096 | 0.351 | 3.345 | 0.014 |
| MCI+ - Young | 0.49 | 4.22 | 0.00056 | 0.537 | 5.374 | <0.0001 |
| AD - Young | 0.69 | 6.43 | <0.0001 | 0.768 | 8.195 | <0.0001 |
| MCI- - Young | 0.05 | 0.41 | 0.99 | 0.145 | 1.390 | 0.79 |
| non-AD - Young | 0.07 | 0.66 | 0.99 | 0.206 | 2.141 | 0.30 |
| CU+ - CU- | 0.37 | 6.66 | <0.0001 | 0.302 | 6.309 | <0.0001 |
| MCI+ - CU- | 0.53 | 10.77 | <0.0001 | 0.488 | 11.456 | <0.0001 |
| AD - CU- | 0.74 | 15.51 | <0.0001 | 0.719 | 17.428 | <0.0001 |
| MCI- - CU- | 0.09 | 1.39 | 0.79 | 0.096 | 1.670 | 0.61 |
| non-AD - CU- | 0.12 | 1.81 | 0.52 | 0.157 | 2.808 | 0.068 |
| MCI+ - CU+ | 0.16 | 2.56 | 0.13 | 0.187 | 3.394 | 0.012 |
| AD - CU+ | 0.37 | 5.85 | <0.0001 | 0.418 | 7.580 | <0.0001 |
| MCI- - CU+ | -0.27 | -3.51 | 0.0082 | -0.205 | -3.028 | 0.038 |
| non-AD - CU+ | -0.25 | -3.24 | 0.019 | -0.145 | -2.154 | 0.30 |
| AD - MCI+ | 0.21 | 3.60 | 0.0061 | 0.231 | 4.594 | 0.0001 |
| MCI- - MCI+ | -0.44 | -5.92 | <0.0001 | -0.392 | -6.113 | <0.0001 |
| mon-AD - MCI+ | -0.41 | -5.68 | <0.0001 | -0.331 | -5.243 | <0.0001 |
| MCI- - AD | -0.65 | -8.89 | <0.0001 | -0.623 | -9.876 | <0.0001 |
| non-AD - AD | -0.62 | -8.80 | <0.0001 | -0.562 | -9.167 | <0.0001 |
| non-AD - MCI- | 0.02 | 0.28 | 0.99 | 0.061 | 0.826 | 0.98 |

ANCOVA was used to compare biomarker levels across groups, adjusting for age and sex. Tukey Honestly Significant Difference (HSD) test was used to account for multiple testing in the post hoc analysis.

*Abbreviations: AD, Alzheimer’s disease dementia; Adj, adjusted; CU−, amyloid-negative cognitively unimpaired; CU+, amyloid-positive cognitively unimpaired; MCI+, amyloid-positive mild cognitive impairment; MCI-, amyloid-negative mild cognitive impairment; p-tau, phosphorylated tau.*

**Table. S3:** ROC analyses.

|  | **p-tau217 ALZpath** | | | | **p-tau217+ Janssen** | | | |  |
| --- | --- | --- | --- | --- | --- | --- | --- | --- | --- |
| **Contrast** | **AUROC (95% Ci)** | **Se/Sp**  **(95% Ci)** | **NPV/PPV**  **(95% Ci)** | **Cut-off** | **AUROC**  **(95% Ci)** | **Se/Sp**  **(95% Ci)** | **NPV/PPV**  **(95% Ci)** | **Cut-off** | **Method** |
| Amyloid PET  + vs - | 0.92  (0.88, 0.95) | 0.82/0.92  (0.73,0.88/0.87,0.95) | 0.88/0.87  (0.82,0.93/0.79,0.92) | 0.42^a^ | 0.92  (0.88, 0.95) | 0.81/0.90  (0.72,0.87/0.85,0.94) | 0.87/0.84  (0.81,0.92/0.77,0.90) | 0.083^a^ | ^a^Previoulsy published by  Ashton *et al* / Therriault *et al* |
| Tau_(Med Temp)_ PET  + vs - | 0.91  (0.87, 0.95) | 0.77/0.93  (0.76,0.85/0.98,0.96) | 0.89/0.85  (0.83,0.94/0.76,0.90) | 0.58 | 0.93  (0.90, 0.96) | 0.84/0.89  (0.75,0.90/0.84,0.93) | 0.92/0.80  (0.78,0.95/0.71,0.88) | 0.09 | Youden |
| Tau_(meta-ROI)_ PET  + vs - | 0.96  (0.94, 0.98) | 1.00/0.71  (0.94,Nc/0.65,0.77) | 1.00/0.48  (0.97,1.00/0.40,Nc) | 0.41 | 0.97  (0.95, 0.98) | 1.00/0.82  (0.84,Nc/0.77,0.87) | 1.00/0.60  (0.98,1.00/0.51,Nc) | 0.09 | Max. Sensitivity |
| Tau_(Neo)_ PET  + vs - | 0.96  (0.94, 0.98) | 0.80/0.95  (0.69,0.89/0.92,0.97) | 0.94/0.84  (0.89,0.97/0.74,0.91) | 0.89 | 0.97  (0.94, 0.99) | 0.86/0.95  (0.83,0.97/0.88,0.95) | 0.96/0.84  (0.94,0.98/0.68,0.92) | 0.12 | Max. Efficiency  (95% Specificity) |
| CI  A+ vs A- | 0.89  (0.82, 0.96) | 0.91/0.83  (0.84,0.96/0.69,0.93) | 0.83/0.91  (0.70,0.93/0.83,0.96) | NA | 0.88  (0.81, 0.95) | 0.85/0.86  (0.75,0.91/0.72,0.94) | 0.74/0.92  (0.61,0.89/0.83,0.96) | NA | Youden |
| CU  A+ vs A- | 0.84  (0.76, 0.92) | 0.73/0.78  (0.54,0.87/0.70,0.86) | 0.91/0.48  (0.82,0.94/0.37,0.71) | NA | 0.86  (0.79, 0.93) | 0.76/0.79  (0.57,0.90/0.71,0.86) | 0.92/0.51  (0.83,0.95/0.39,0.74) | NA | Youden |
| CI A+T_(meta-ROI)_+  vs CI other | 0.91  (0.86, 0.96) | 0.87/0.81  (0.75,0.89/0.70,0.89) | 0.89/0.77  (0.79,0.94/0.65,0.89) | NA | 0.92  (0.88, 0.96) | 0.92/0.80  (0.82,0.97/0.69,0.88) | 0.93/0.77  (0.84,0.96/0.65,0.92) | NA | Youden |
| A+T_(meta-ROI)_+  vs all other | 0.95  (0.93, 0.97) | 0.96/0.86  (0.87,0.99/0.81,0.90) | 0.99/0.63  (0.96,0.99/0.54,0.93) | NA | 0.96  (0.94, 0.98) | 0.94/0.89  (0.85,0.98/0.84,0.93) | 0.98/0.68  0.95,0.99/0.58,0.91) | NA | Youden |
| A- vs A+ Tau_“low”_* | 0.87  (0.78, 0.96) | 0.78/0.87  (0.56,0.92/0.81,0.91) | 0.96/0.45  (0.91,0.98/0.34,0.73) | NA | 0.92  (0.87, 0.97) | 0.95/0.79  (0.78,0.99/0.72,0.84) | 0.99/0.37  (0.95,0.99/0.29,0.96) | NA | Youden |
| A+  T_(Neo)_- vs T_(Neo)_+ | 0.89  (0.83, 0.95) | 0.74/0.92  (0.61,0.84/0.82,0.97) | 0.76/0.92  (0.63,0.92/0.80,0.95) | NA | 0.91  (0.86, 0.96) | 0.87/0.81  (0.76,0.94/0.69,0.90) | 0.84/0.84  (0.72,0.92/0.72,0.92) | NA | Youden |

All AUROCs above were compared between the two assays and none of them had a statistical difference using the Delong’s test (two-tailed).

* This analysis contrasted all participants that were Amyloid PET- with participants that were Amyloid PET+ and Tau_(Med Temp)_+ (participants T_(Neo)_ + were excluded). Threshold SUVR for these classifications are reported in the Methods.

*Abbreviations: A+, Amyloid PET positive; A-, Amyloid PET negative; AUROC, Area under the receiver operating characteristic curve; Ci, Confidence interval; CI, Cognitive impairment; CU, Cognitively unimpaired; Med. Temp., Medial Temporal; NA, Not applicable; Nc, not computed; Neo, Neocortical; NPV, Negative predictive value; PPV, Positive predictive value; p-tau, Phosphorylated tau; ROC, Receiver operating characteristic curve; ROI, Region of interest; Se, Sensitivity; Sp, Specificity.*

***IMPORTANT NOTE:***

The cut-off thresholds provided in the table above are presented for the sole purpose of comparing biomarker assays. As of this publication date, these cut-offs have not undergone validation. Caution is advised when considering their application in future studies.

**Figure S1:** Box-plot showing plasma p-tau 217 levels across groups displaying raw values on pg/mL

The boxplots depict the median (horizontal bar), 25th to 75th percentiles (hinges) and whiskers indicate 10th and 90th percentiles. Group comparisons (presented in the Supplementary material) were computed with a one-way ANCOVA adjusting for age and sex. Tukey honestly significant difference (HSD) test was used for the post hoc pairwise comparisons. The * indicates, for each biomarker, the groups that are significantly different from CU- group (see Table S2 for all group comparisons).

**Figure S2:** Correlation of plasma p-tau217 assays with CSF p-tau217.

Scatter plot showing the correlation between plasma p-tau217 biomarkers with CSF p-tau217 (n=178). The line represents the locally estimated scatterplot smoothing (LOESS; span= 1) regression.

**Figure S3:** **Correlation of plasma p-tau217 assays with amyloid-PET uptake in amyloid PET positive and amyloid PET negative groups separately.** The plots show Spearman rank correlations between p-tau217 assays and amyloid PET in amyloid negative participants (top plots in blue) and in amyloid positive participants (bottom plots in yellow). The line represents the locally estimated scatterplot smoothing (LOESS; span= 1) regression.

**Fig S4: Correlation between plasma p-tau217 and tau PET in cognitively impaired amyloid PET positive individuals.** Spearman correlation of plasma p-tau217+ Janssen and plasma p-tau217 ALZpath with neocortical tau PET in amyloid PET positive individuals with MCI (orange) and AD dementia (red). Plasma p-tau217+ Janssen had a strong association with temporal neocortical tau PET (*ρ_neocort_*.=0.72, *P*<0.0001), as did the ALZpath p-tau217 assay (*ρ_neocort_.*= 0.61, *P*<0.0001). Comparison of correlation coefficients in this subgroup revealed a slightly higher association with tau PET for the Janssen p-tau217+ assay (*P*=0.044; 95% CI = 0.004 – 0.23). The line represents the locally estimated scatterplot smoothing (LOESS; span= 1) regression.

**Figure S5: Individual-level agreement between plasma p-tau217 assays.**

Scatter plot showing the distribution of plasma p-tau217 concentrations across assays and cut-offs. The dashed lines indicate the cut-off values for of each of the plasma p-tau217 assays to indicate amyloid PET status (A+) and neocortical tau PET status (T+_(Neo)_). The shaded areas represent the range of values in which individuals would be classified as amyloid PET negative (green), amyloid PET positive (orange) as well as amyloid PET positive and neocortical tau PET positive (red) based on the plasma assays cut-offs. The values displayed within the plot show the percentage (%) agreement between plasma assays. The dots are coloured according to the individual status on the PET imaging classification, as presented in the legend in the figure.

**Figure S6: Correlation of annual change of plasma p-tau217+ and plasma p-tau217.** Spearman correlation tests of p-tau217 biomarkers % of change per year (left plot) and delta change per year (right plot). The line represents the locally estimated scatterplot smoothing (LOESS; span= 1) regression.
